# Teaching extent and military service improve undergraduate self-assessed knowledge in disaster medicine: An online survey study among Swedish medical and nursing students

**DOI:** 10.3389/fpubh.2023.1161114

**Published:** 2023-03-31

**Authors:** Yohan Robinson, Luca Ragazzoni, Francesco Della Corte, Johan von Schreeb

**Affiliations:** ^1^Institute for Clinical Sciences, Sahlgrenska Academy, Gothenburg University, Gothenburg, Sweden; ^2^CRIMEDIM—Center for Research and Training in Disaster Medicine, Humanitarian Aid, and Global Health, Università del Piemonte Orientale, Novara, Italy; ^3^Department of Global Public Health, Center for Research on Health Care in Disasters, Karolinska Institute, Stockholm, Sweden

**Keywords:** civil defense, disaster medicine, emergency medical services, military medicine, undergraduate medical education, nursing students, medical students

## Abstract

**Background:**

The purpose of this study was to identify the possible needs for undergraduate disaster medicine education in Sweden and to make informed recommendations for the implementation of disaster medicine content in medical and nursing schools in Sweden.

**Methods:**

An online survey was distributed to undergraduate medical and nursing students through the directors of all medical and nursing programs at Swedish universities. The survey contained demographic questions, as well as questions about the amount of disaster medical education and previous experience with rescue, police, or military services. The final survey page contained self-assessments of disaster medical knowledge. Comparative statistics were applied between nursing and medical students, those with previous military service, and those without, as well as between universities.

**Results:**

A total of 500 medical and 408 nursing students participated in this study. A median of 2 h of disaster medicine education was provided to senior medical students and 4 h was provided to senior nursing students. Senior medical students scored their disaster medical knowledge lower than nursing students (*t*-test, *p* < 0.001). A proportion of 1% had served in rescue services or police, and 7% of the participants had a history of military service, of which 67% served in a medical role. Those who had served in rescue services, police, or the armed forces had a higher self-assessed disaster medical knowledge base than those who had not (*p* < 0.007 and *p* < 0.001, respectively).

**Conclusion:**

Most medical and nursing students in this study rated their disaster medical knowledge as insufficient. The correlation between the amount of disaster medical education and self-assessed disaster medical knowledge should influence and help direct Swedish educational policies.

## 1. Introduction

Disaster medicine has been defined as the science and practice of the analysis and development of the methodology necessary to handle situations where available resources are insufficient in relation to the immediate need for healthcare (1). It shares content with other specialties such as military medicine, emergency medicine, and public health, and covers the entire disaster management cycle (2).

In Sweden, storms are the most common natural disaster with seven storms annually, followed by two epidemics, one flood, one heat wave, and one wildfire disaster annually (3). The main disaster death tolls in the country are caused by pandemics, maritime disasters and fires. As the number of individuals exposed to disasters is increasing owing to population growth, increasing numbers of armed conflict, and climate change, there is a global need for medical doctors and health practitioners to become familiar with disaster medical principles and understand the dynamics of healthcare needs in disaster relief operations (4, 5). Therefore, professional disaster medical education and training have been listed among the priority 1 goals of the Sendai Framework for Disaster Risk Reduction (6).

Training typically involves instruction and practice aimed at reaching a particular level of competence or operative efficiency, where skill acquisition is the main ingredient, while education delivers conceptual insight, explanatory principles, and justificatory or interpretative frameworks, and develops critical reflectiveness and autonomy of judgment (7, 8). The required amount of training or education and skills in disaster medicine depends on the student’s future role in the disaster response system (9).

Multiple disaster medical curricula for healthcare professionals have been developed at undergraduate and postgraduate levels, but a global consensus on teaching content and methodology, as well as on curriculum implementation, is lacking (10). In Germany, an undergraduate curriculum was developed consisting of 14 2-h modules containing educational lectures, simulation exercises, and practical training (11), but 9 years later, the implementation of this curriculum in medical schools was poor (12).

Insufficient undergraduate disaster medicine education and training has been reported in many countries. Only 17% of American medical students believe that they are receiving adequate education and training for natural disasters, and more than 30% of United States med schools do not offer disaster medicine curricula (13). In Germany, only 12% of medical students are taught disaster medicine content at their institution, but more than 30% attend courses outside their university (i.e., Red Cross) (12). In the Netherlands, medical students rate their knowledge and capability to deal with CBRN incidents at 20% (14), while medical students from the Belgian Armed Forces rate around 40% (15).

Disaster medical education in medical and nursing schools has distinct political implications. For instance, after the Fukushima nuclear power plant accident, the Japanese undergraduate radiation disaster medical curriculum for medical students changed from 6 to 82 h (16). In Sweden, the COVID-19 pandemic and the Ukraine invasion war increased the political pressure on public health and civil defense agencies to increase preparedness for pandemics and armed conflict. However, recently, undergraduate disaster medical education in Sweden has been reported to be insufficient (17). Only half of Swedish nursing schools have trauma and disaster medical content in their undergraduate curricula, and only 16% of these include undergraduate disaster exercises (18).

This finding was surprising, as Sweden has had well-functioning disaster medical undergraduate education and training since 1974 (19). All medical students were given a 1-week course in disaster medicine, which included both lectures and practical training. In practical exercises, students learn to cooperate with police and civil protection agencies during a disaster (20). This course was discontinued in the early 2000s, and Swedish universities downprioritized disaster medicine in favor of other medical topics (17).

The current undergraduate exposure of nursing and medical students to disaster medical content remains unknown. Furthermore, the Swedish National Board of Health and Welfare recommends an increase of medical and nursing students’ undergraduate teaching (21), but reliable data on what extent of disaster medical education for medical and nursing students are sufficient is unavailable. Beyond that, the effect of practical experience with contingency preparedness within uniformed services prior to entering medical or nursing programs has not been researched, yet.

This study has the ambition to address the above knowledge gaps by assessing:

1. To what extent Swedish nursing and medical students are similarly exposed to disaster medicine in their respective curricula; and
2. Whether previous rescue, police, or military service improves undergraduate self-reported knowledge of disaster medicine principles.

## 2. Materials and methods

### 2.1. Study design

This is a cross-sectional, online survey study among medical and nursing students in Sweden to gather information on the amount of education and training related to disaster medicine that future physicians and nurses are exposed to. This study was reported according to the Checklist for Reporting of Survey Studies (CROSS) (22).

### 2.2. Data collection methods

Since longer surveys have a risk of reduced response rates, we designed a survey where only a few key questions were included. This was to prioritize the number of responses over information depth. The full Swedish survey is available in Supplementary 1.

The survey asked respondents about their exposure to disaster medicine during their undergraduate studies.

The target population of this study included medical and nursing students at various stages during their training. Potential respondents were eligible for the survey only if they could answer positively to the first survey question: “Are you a medical student, a nursing student, a physician, or a nurse in Sweden?” It is estimated that a total of 8,441 students were enrolled in medical programs while 15,538 in nursing schools at the time of the study.

### 2.3. Initial questionnaire development

The survey consisted of five sections: demography, undergraduate and postgraduate education and training, previous civil/military service, and disaster medical knowledge self-assessment.

The Swedish National Council for Disaster Medicine, consisting of the directors of Swedish research centers for disaster medicine, informed the choice of survey topics on how to assess and improve the undergraduate and postgraduate disaster medicine education of physicians and nurses.

The final page of the survey consisted of a validated self-assessment of the knowledge base in disaster medicine by Wunderlich et al. (12). These statements are based on the disaster medical curriculum proposed by Pfenninger et al. (11) The 13 statements are as follows:

1. I know the terminology of disaster medicine, its legal aspects, and its classification.
2. I know the organization and leadership to deal with a large number of casualties.
3. I know how to deal with a number of patients that exceeds the normal capacity of the medical system.
4. I know the basics of alarm and evacuation of hospitals in the case of an external disaster.
5. I can evaluate the practicability of medical disaster response using practical examples.
6. I can use the principles of triage in pre-clinical and clinical settings.
7. I know the principles and the procedures of a necessary evacuation.
8. I know the basics of primary healthcare under disaster circumstances (life-saving procedures, shock treatment).
9. I know the basics of specific healthcare under disaster circumstances (surgical emergency procedures and procedures after thermal damage).
10. I am aware of medical management after accidents involving radioactive materials and their decontamination.
11. I am aware of the management of transportation of hazardous materials and accidents, as well as the management of mass intoxication with chemicals and decontamination.
12. I know the basics of ethical actions in disaster medicine and quality management in disaster response.
13. I understand the basics of diseases triggered by disaster situations and the actions of psychosocial support in disaster situations.

The answers were recorded on a Likert scale from 1 (“I strongly disagree”), 2 (“I disagree”), 3 (“Neutral”), 4 (“I agree”), to 5 (“I strongly agree”).

#### 2.3.1. Validity and reliability

The validity of the survey items was assessed in consecutive stages by requesting individual experts to evaluate the content relevance and simplicity of the individual items and the entire set of items (questionnaire) as a tool, followed by iterative loops of consensus panel revisions. Finally, the validated questionnaire was pre-tested on a sample from the target population.

The consensus panel consisted of four main authors of this paper: three professors in disaster medicine and one senior lecturer in orthopedics.

The expert panel consisted of six members from the Swedish National Council of Disaster Medicine: One professor in emergency medicine, one professor in surgery, one senior professor, one associate professor, and two researchers in nursing and prehospital emergency care. The expert panel was selected based on their experience and current responsibilities in Swedish disaster medical education and training. The validation process was performed in three consecutive assessment stages. In the first two stages, experts assessed the questions individually and as a questionnaire tool with respect to their content relevance and simplicity. A marked text file was used for assessment and panel review. This was followed by a revision of the questionnaire by the consensus panel. In the final stage of the validation process, the Scientific Committee of CRIMEDIM—Center for Research and Training in Disaster Medicine, Humanitarian Aid, and Global Health, Università del Piemonte Orientale (Novara, Italy), scrutinized the questionnaire and study design, followed by a final revision by the consensus panel.

#### 2.3.2. Pre-testing

The questionnaire was pre-tested with 20 students in the eighth semester of the medical program at Gothenburg University, Sweden, regarding their questionnaire user experience. The respondents received a QR code for the web survey immediately after their 2-h disaster medical lecture and were requested to offer their opinions on the overall questionnaire tool separately after completing the survey. All 20 students completed the survey in 4 min. No complications were recorded during the pre-test.

### 2.4. Sample characteristics

#### 2.4.1. Eligibility

The inclusion criteria were

1. Medical student or nursing student
2. Enrolment at a Swedish university

Exclusion criteria were

1. Age below 20 years or above 70 years
2. Enrolment at a university outside of Sweden

#### 2.4.2. Sample size

According to the Swedish Higher Education Authority 8,441 medical students and 15,538 nursing students were registered at Swedish universities in spring 2022. The required sample size for a 95% CI and a 5% margin of error was 384 based on the Cochran sample size formula for categorical data (8).

### 2.5. Survey administration

To reach medical and nursing students, the program directors of every faculty of medicine and nursing in Sweden were asked to distribute the online survey through a group email to medical and nursing students. Three of the six medical colleges and six of the 25 nursing colleges agreed to participate in the study. Two of the six medical colleges did not allow online surveys to be distributed to their students. Nineteen nursing program directors did not respond to the survey.

The survey was conducted using SurveyMonkey. The survey was conducted between August 15th and September 30th, 2022.

### 2.6. Ethical considerations

This study was approved by the Swedish Ethical Review Authority (no. 2022-01800-01).

As the data for this study are collected from an online survey harm of integrity through inappropriate survey anonymity and confidentiality are identified ethical risks. This study collects for instance information on gender, birth year, region and profession. Since the number of medical and nursing students is high in Sweden, with 8,441 medical students and 15,538 nursing students in spring 2022, the possibility of tracing anonymized data to individuals is very low. To additionally protect the data from unauthorized access, we used an established survey provider with a two-level password protection.

This survey study has a relatively low risk of physical or psychological harm, pain, or discomfort, as it comprises of a collection of information about the study person’s educational background with regard to disaster medicine. The self-assessment may cause discomfort as it may reveal the own knowledge gaps, but the risk of this causing physical or psychological harm is very low.

The gain for the individual participant is the resulting improvement of disaster medical education, which will strengthen the national disaster resilience and the individual’s safety.

### 2.7. Statistical analysis

For data preparation, we exported the dataset from SurveyMonkey to Microsoft Excel and for statistical analysis we used R Commander (version 4.2.1) and RStudio (version 2022.07.2 + 576).

#### 2.7.1. Respondent characteristics and disaster medical education

The following descriptive data were collected: age, sex, region/county, university, year of study, previous military service, previous rescue, or police service employment. The descriptive data are presented in a separate table. To map the amount of disaster medical content each student receives, we calculated the mean and median hours of disaster medical education received during the current study, presented with standard deviation and range. The differences between universities were tested using t-tests.

#### 2.7.2. Disaster medical knowledge base and effects of military or civilian prehospital service

Regarding the students’ knowledge base in disaster medicine, median values, 25 and 75% quartiles are presented for each item, and mean and standard deviation for all items together.

An analysis of covariance was conducted with five demographic variables as independent variables and mean self-assessed disaster medical knowledge as the dependent variable. Age, sex, type of program, military service, and rescue/police experience were the five independent variables. These were tested using two-sided ANOVA models for interaction. The model with a significant contribution to the variance of the dependent variable is presented with F-scores and *p* values. Furthermore, we performed linear regression of the model response and used standardized regression coefficients as direct measures of sensitivity.

#### 2.7.3. Non-response error

The number of non-responders was not available in this survey, as we had no information on how many medical and nursing students had received the invitation to participate. This indicates that our sample was not random. We were able to generate results for those who responded, but the results were not generalizable. Therefore, we treat the results as qualitative, indicative, and hypothesis-generating. Partial responders were analyzed and compared to full respondents concerning demographics, such as gender, nursing or medical program, disaster medical content, and undergraduate exercises, using the χ^2^-test.

## 3. Results

### 3.1. Respondent characteristics

A total of 908 students participated in the study. Respondents required a mean time of 3 min and 5 s to complete the questionnaire. A total of 82% replied to the complete questionnaire. The question “Do you have a profession in rescue services or police?” was not responded to by 18% (162 of 908), the question “Did you participate in disaster medicine courses with civilian organizations?” was not responded to by 14% (131 of 908), and the question “Did you serve in the Swedish armed forces?” was not responded to by 9% (83 of 908). The disaster medical knowledge self-assessment was not responded to by 18% (160 of 908).

Partial respondents did not differ significantly from respondents regarding gender (χ^2^-test, *p* = 0.44), medical or nursing program (χ^2^-test, *p* = 0.73), or undergraduate disaster exercises (χ^2^-test, *p* = 0.42) but reported to a higher proportion that they did not have any disaster medical education (χ^2^-test, *p* = 0.01; Table 1).

**Table 1:** Number of full respondents vs. partial respondents and analysis of gender, disaster medical education and participation in simulation exercises presented with *p* values of χ^2^-test.

		Gender		Disaster medical education			Exercises	
	*n*	Female	Male	Compulsory	Elective	None	Yes	No
**Full respondents**	750	534	211	107	65	578	167	528
Medical students	411	249	159	26	61	324	61	320
Nursing students	339	285	52	81	4	254	106	208
**Partial respondents**	158	107	49	7	5	90	16	64
Medical students	89	51	38	3	5	51	8	36
Nursing students	69	56	11	4	0	39	8	28
*p*(χ^2^-test)	0.72	0.44		0.01			0.42	

### 3.2. Demographics

Of the 908 participants, 500 were medical students (age, 26 ± 6 years; 60% female) and 408 nursing students (age 28 ± 8 years, 84% female). Medical students were younger (*t*-test, *p* < 0.001), and nursing students had a larger proportion of females (χ^2^-test, *p* < 0.001).

There were 13% (59 of 470) medical students each in the first (59 of 470), second (59 of 470), and third (63 of 470) semesters. Meanwhile 7% (35 of 470) were in the fourth, 4% (20 of 470) were in the fifth, 5% (35 of 470) were in the sixth, 9% (43 of 470) were in the seventh, 13% were in the eighth, 5% were in the ninth, 9% were in the tenth, and the final semester had a total of 9%. Nursing students were split into 16% (61 of 383) in the first semester, 18% (69 of 383) in the second semester, 14% (55 of 383) in the third semester, 18% (68 to 383) in the fourth semester, and 17% each in the fifth (66 of 383) and final (64 of 383) semesters.

Participants were representative of Sweden’s geography. Approximately 39% of participants were residents of the western Swedish region Västergötland (353 of 908), followed by 27% from the eastern region Stockholm (243 of 908), 12% from the southern regions Skåne (68 of 908), and Blekinge (40 of 908), and 11% from the northern region Västerbotten (100 of 908).

At Gothenburg University, 49% of medical students studied. At Karolinska Institute, 47% studied, 2% studied at Uppsala University, 1% at Linköping University, and less than 1% at Umeå University. Medical colleges at Örebro University and Lund University chose not to participate in this study. A total of 37% of nursing students studied at Umeå University, 31% at Gothenburg University, 13% at Blekinge Technical University, 11% at Lund University, 5% at Linné University, and 1% at Malmö University. Nineteen other nursing colleges chose not to participate in the study.

In both medical and nursing colleges, the most common reason for non-participation was internal university rules which prohibited the distribution of external questionnaires.

Medical and nursing students did not differ significantly with regard to experience of prior military service (8 vs. 5%, χ^2^-test, *p* = 0.10). Nursing students had a higher proportion of experience with rescue or police services than medical students (2 vs. 0.5%, χ^2^-test, *p* = 0.02). The demographics are summarized in Table 2.

**Table 2:** Summary of the demographics of the included medical and nursing students.

Group (n)	Age years (±SD)	Male *n* (prop)	Female *n* (prop)	Military service *n* (prop)	Rescue or police service *n* (prop)	Disaster medical education hours (±SD)
*n*	908	260	641	58	10	908
Medical students	26	197	300	38	2	5
(500)	(±6)	(40%)	(60%)	(8%)	(0.5%)	(±25)
Nursing students	29	63	341	20	8	11
(408)	(±8)	(16%)	(84%)	(5%)	(2%)	(±59)

### 3.3. Exposure to disaster medical education

Medical students received a mean of 5 ± 25 h of disaster medical education. Nursing students received a mean of 11 ± 59 h of disaster medical education (*t*-test, *p* = 0.16). Approximately 16% (69 of 426) of medical students and 32% (114 of 361) of nursing students have participated in disaster response simulation exercises (χ^2^-test, *p* < 0.001).

**Figure 1:**
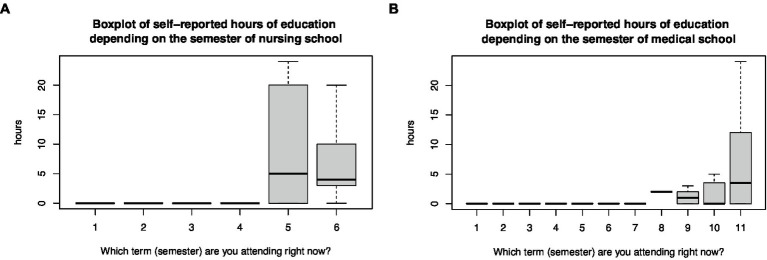
Boxplot of the self-reported hours of education in disaster medicine depending on the semester of **(A)** nursing and **(B)** medical programs.

The data analysis according to semester revealed that medical students received their disaster medicine education from the eighth semester, and nursing students from the fifth semester (Figure 1). Among these senior students, disaster medical exercises were included in 20% (33 of 161) of the medical programs and in 47% (60 of 128) of the nursing programs (χ^2^-test, *p* < 0.001).

### 3.4. Self-assessed disaster medical knowledge

**Figure 2:**
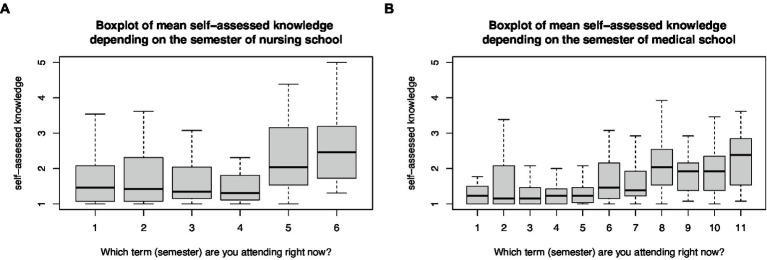
Box plot of mean self-assessed disaster medical knowledge base depending on the semester of **(A)** nursing and **(B)** medical programs.

The mean self-assessed knowledge score increased linearly from the first semester to the final semester for both medical (*r* = 0.35, *p* < 0.001) and nursing students (*r* = 0.34, *p* < 0.001; Figure 2). The increase in mean self-assessed knowledge did not differ between junior medical students up to the seventh semester and senior medical students from the eighth semester (*t*-test, *p* = 0.23). However, it did differ between junior nursing students below the fourth and senior nursing students above the fifth semester (*t*-test, *p* < 0.001). These were semesters when disaster medicine was commonly introduced to students. Therefore, a subgroup analysis of the disaster medical knowledge base was performed among 165 senior medical students in the 8–11th semesters and 130 senior nursing students in the fifth and sixth semesters (Table 2). Senior medical students received a median of 2 h of disaster medical education and senior nursing students received a median of 4 h (*t*-test, *p* = 0.16).

**Figure 3:**
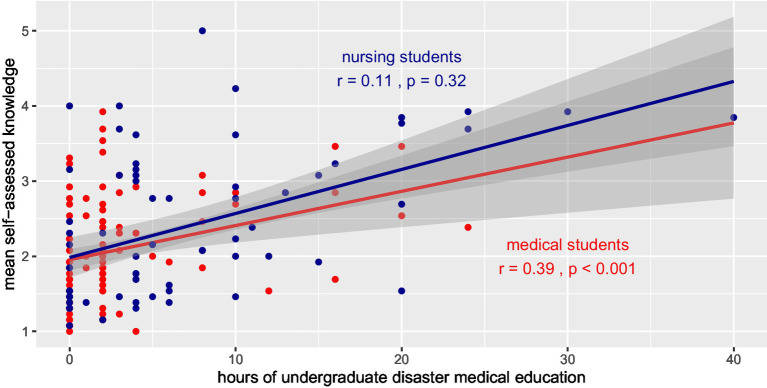
Correlation of hours of education and mean self-assessment of disaster medical knowledge among senior medical (red) and nursing (blue) students presented with linear trend lines.

The number of hours of disaster medical education correlated with the self-assessed knowledge base of senior medical students (*r* = 0.39, *p* < 0.001), but not with senior nursing students (*r* = 0.11, *p* = 0.32; Figure 3). The correlation allowed us to predict that approximately 20 h of disaster medical teaching were necessary to reach a “neutral” level of self-assessed knowledge. The linear trend predicts that a minimum of 40 h of teaching is necessary to allow medical students to agree to the 13 statements in the survey.

**Figure 4:**
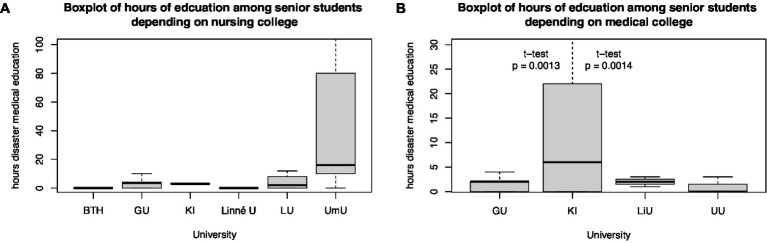
Boxplot of hours of education among senior **(A)** nursing and **(B)** medical students depending on their university.

Self-reported hours of disaster medical teaching differed between colleges. Among nursing colleges, Umeå University, and among medical colleges, Karolinska Institute had the highest number of hours in disaster medicine education (Figure 4).

**Figure 5:**
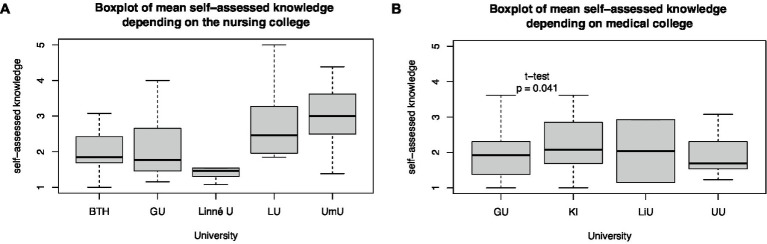
Differences in mean self-assessed knowledge depending on the **(A)** nursing and **(B)** medical university.

Self-assessed disaster medical knowledge differs among universities. Gothenburg University’s senior medical students had lower self-assessed disaster medical knowledge than the Karolinska Institute of Medical Students from the 8 to 11th semesters (*t*-test, *p* = 0.04). Differences between other medical colleges could not be estimated because of the small sample size. Even between the nursing colleges of Umeå and Gothenburg University (*t*-test, *p* < 0.001), Umeå and Blekinge Technical University (*t*-test, *p* < 0.01), and Umeå and Linné University (*t*-test, *p* = 0.03), differences in self-assessed knowledge of senior nursing students in semesters 5 and 6 were identified (Figure 5).

### 3.5. Differences between medical and nursing students

**Figure 6:**
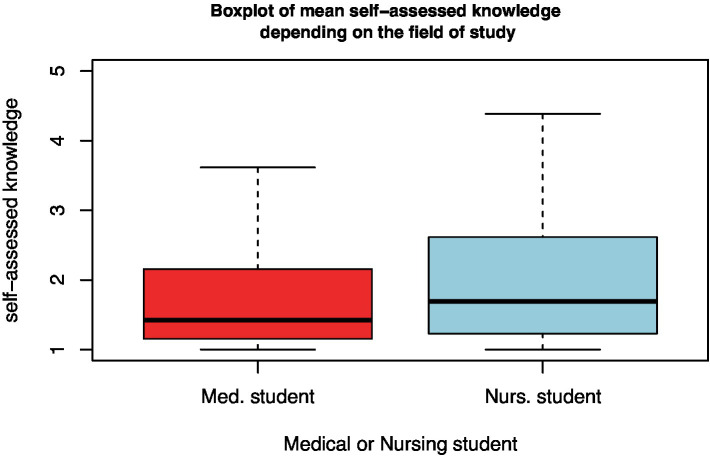
Boxplot of the mean self-assessed disaster medical knowledge of medical and nursing students.

Senior medical students scored their disaster medical knowledge lower than nursing students both for each individual question (Table 3) and as a difference in mean self-assessed knowledge (*t*-test, *p* < 0.001; Figure 6).

**Table 3:** Summary of self-assessment of disaster medical knowledge by senior medical (semesters 8–11) and nursing students (semesters 5 and 6) on a Likert scale [1 (“I strongly disagree”), 2 (“I disagree”), 3 (“Neutral”), 4 (“I agree”), to 5 (“I strongly agree”)], presented as median with 25 and 75% quantiles.

Self-assessment of disaster-medical knowledge base		Medical student			Nursing student		
Statement		25%	Median	75%	25%	Median	75%
1.	I know the terminology of disaster medicine, legal aspects, as well as the disaster classification.	1	2	3	1	2	4
2.	I know the organization and leadership to deal with a large number of casualties.	1	1	1	1	1	2
3.	I know how to deal with a number of patients, which is exceeding the normal capacity of the medical system.	1	2	3	1	2	4
4.	I know how the basics about alarm and evacuation of hospitals in case of an external disaster.	1	2	3	1	2	4
5.	I can evaluate the practicability of medical disaster response in practical examples.	1	2	2	1	2	3
6.	I can use the principles of Triage in a pre-clinical and clinical setting.	1	1	2	1	2	3
7.	I know the principles and the procedures of a necessary evacuation.	1	2	3	2	3	4
8.	I know the basics of primary health care under disaster circumstances (life-saving procedures, treatment of shock).	2	3	4	2	4	4
9.	I know the basics of specific health care under disaster circumstances (surgical emergency procedures, procedures after thermal damage).	1	1	2	1	2	3
10.	I am aware of the medical management after accidents with radioactive materials and the decontamination of these.	2	3	4	2	3	4
11.	I am aware of the management of transports of hazardous material and accidents as well as the management of mass intoxications with chemicals and decontamination.	2	2	3	1	2	3
12.	I know the basics of ethical action in disaster medicine and the quality management in disaster response.	1	2	3	2	3	4
13.	I know the basics of diseases triggered by disaster situations and the actions of psychosocial support in disaster situations.	1	1	2	1	1	2

### 3.6. Previous military service, civilian rescue, or police employment and disaster medical knowledge

A proportion of 7% (58 of 825) of the participants had a history of military service, with a median start of military service in 2016 (mean 2013, range 1992–2022). Of those who served in the military, 67% (39 of 58) served in a medical role.

**Figure 7:**
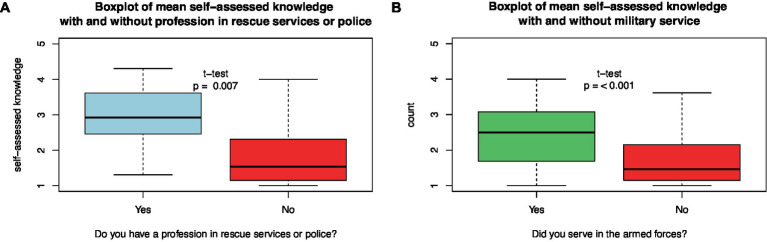
Differences in mean self-assessed disaster medical knowledge between **(A)** those who had served in rescue/police services or not and **(B)** those who had served in the armed forces or not.

Students with previous military service had higher self-assessed disaster medical knowledge than those without military experience (*t*-test, *p* < 0.001; Figure 7). This difference was also found in the subgroup of senior students, where 4.5% (13 of 287) had served in the military (*t*-test, *p* = 0.003).

About 1.3% (10 of 746) of students reported having worked in civilian rescue or police services. Students with rescue or police service experience had higher self-assessed disaster medical knowledge than those without military experience (*t*-test, *p* = 0.007; Figure 7). In the subgroup of senior students, only 0.8% (2 of 255) had experience with rescue or police services (*t*-test, *p* = 0.42).

As there were more men than women entering military service, we tested the interaction between gender and military service. This variation could not be explained by the interaction between gender and military service (sum sq. = 0.26, *p* = 0.52). The type of program, whether nursing or medical students [*F*(1, 248) = 15.959, *p* < 0.001], was significantly related to the mean self-assessed disaster medical knowledge. There was also a significant effect of previous military service after controlling for the effect of program type [*F*(2, 248) = 9.891, *p* = 0.002].

## 4. Discussion

To the best of our knowledge, this is the first study to self-assess disaster medical knowledge among Swedish medical and nursing students. Among those that replied to our survey we found that the amount of disaster medical education differed between universities, and that more hours of education were correlated with higher values of self-reported disaster medical knowledge. In addition, we found that among those who responded to the survey, being enrolled in a nursing program, and having concluded that previous military service, had a positive effect on higher self-assessed disaster medical knowledge.

### 4.1. Limitations

The major limitation of this study is related to non-response bias, as it is unknown how many medical and nursing students received the invitation to participate. About 6% (500 of 8,441) of all medical students and 3% (408 of 15,538) of all nursing students participated in this questionnaire. This does not give a response rate, but an estimate of how large part of all students that answered. Most universities and colleges did not respond to the questionnaire invitation. Therefore, the survey population was not representative of the entire population. The sample size was sufficient to perform subgroup analyses with statistical power for the primary endpoint self-assessed knowledge and secondary endpoints such as nursing or medical program, sex, and military service.

Additionally, a selection bias is possible when students who have an interest in disaster medical topics are more inclined to reply. We attempted to minimize this bias by shortening the survey so that it could be completed in 5 min.

Another potential bias is related to multiple entries from the same individual. To prevent this, the online survey platform used in this study did not accept multiple entries from the same IP address. An anonymous online survey, however, cannot be 100% free of such manipulations.

As the survey was retrospective, recall bias should be assumed, in which memories of disaster medical education efforts may have been lost or strengthened.

### 4.2. Interpretations

#### 4.2.1. Disaster medical education and self-assessment

In the 1990s, all medical students in Sweden were given a 1-week course in disaster medicine during the fourth year of university studies (20). About 25 years later, we found the amount of disaster medical education to be reduced to a median of 2 h.

In 2022, the Swedish Ministry of Health instructed the National Board of Health and Welfare to develop national plans for disaster medicine education and exercises (RU S2021/02922). The National Board of Health and Welfare responded that disaster medicine must be taught in undergraduate programs to professionals in the healthcare sector (21). Undergraduate disaster medical education was recommended as the basis for postgraduate training and exercises in disaster medicine and included 4 days of education. As Swedish universities are independent authorities, the Ministry of Health has no formal authority over universities, complicating the implementation of the undergraduate educational plan. Our survey revealed significant differences between Swedish universities, indicating a lack of nationwide consensus on disaster medical content in medical and nursing programs. A nationwide consensus, including universities with medical and nursing programs and medical and nursing professional societies, is necessary if disaster medicine is to be given higher priority.

Even though learning is not only dependent on the number of hours spent in class but also on the teaching method, we found a correlation between self-assessed disaster medical knowledge and the hours of disaster medical education assigned to medical and nursing students. Our data predict that a minimum of 40 h of disaster medical teaching would be necessary for medical students to agree on the disaster medical knowledge statements in the questionnaire (Figure 3). Obviously, the 1-week course that Sweden offered during the late 1990s (20) could be a reasonable educational format for reaching an acceptable level of knowledge of basic disaster medicine. The recommendation of the National Board of Health and Welfare to increase current undergraduate teaching in disaster medicine to four full days is a step in this direction (21).

Insufficient disaster-related medical content in medical and nursing programs has been described in multiple countries. In the Netherlands, no universities offer disaster medicine training in their curricula. In a survey of 999 Dutch senior medical students, knowledge assessment revealed serious gaps, leading to decisions with possible life-threatening consequences for themselves, colleagues, and patients (14). In Germany, only 12% of 992 surveyed medical students have attended disaster medical courses, of which 54% are elective courses (12). Because they used the same statements for knowledge assessment as in our study, they can be compared with our study results. German students rated the median knowledge of disaster medicine organizations for mass casualty incidents higher than Swedish students. In addition, Swedish students rated the median knowledge of management after CBRN accidents higher than German students did.

#### 4.2.2. Differences between medical and nursing students

The survey revealed that the nursing programs that participated in the study spent more hours on disaster medicine content and disaster medical simulation exercises than did the participating medical programs (Figure 4) Therefore, it is not surprising to find that the self-assessment of senior nursing students was higher than that of medical students (Figure 6).

Few studies have compared the disaster medical knowledge of different healthcare professions, and most have focused on disease outbreak management. An American study among 579 junior medical, nursing, and pharmacy students investigated their knowledge of and willingness to assist in a contact and respiratory transmittable disease outbreak context (23). They found medical students to be more afraid of their health than nursing students but also the most willing to work. Pharmacy students demonstrated the lowest willingness to work. Medical students scored the highest on the knowledge assessment.

During the COVID-19 pandemic, 1,021 Romanian medical and nursing students were surveyed for disaster medical skills and preparedness to efficiently protect against COVID-19 infection (24). They found that nursing students scored higher in both theoretical and practical preparedness compared to medical students (*p* < 0.001) and discussed the more practical approach in nursing programs compared to medical programs as the underlying reason for these differences.

#### 4.2.3. Military and civil service and disaster medical knowledge

Since the 1990s, when all male physicians received military training and were part of a medical reserve (20), Sweden abandoned the concept of a conscript army and chose the professional armed forces concept. Our questionnaire revealed that by 2022, only 8% of medical students and only 5% of nurses had military training. Furthermore, we identified that, in addition to the educational program, previous military service was the most reliable predictor of higher self-assessed disaster medical knowledge.

An online survey among Belgian military and civilian medical students found that military medical students scored higher in self-assessed disaster medical knowledge and capability in all investigated fields, except influenza pandemic management and care (15).

As in our study, most medical and nursing students who served in the military served in a medical position; one could assume that the military service supplied medical and nursing professionals with more competent and prepared students. As the Swedish government decided to rebuild the Conscript Army, we assume that this will improve the disaster preparedness of medical and nursing students.

## 5. Conclusion

### 5.1. The Swedish undergraduates rate their disaster medicine knowledge low

Most medical and nursing students enrolled in this study rated their disaster medical knowledge as insufficient. Therefore, it is recommended that Swedish universities address this gap in medical and nursing curricula to supply a competent workforce to national disaster preparedness organizations. We urge medical and nursing faculties not to take responsibility for disaster healthcare education lightly, and to strengthen disaster health content in their curricula. As this requires more disaster medical university teachers, research programs aimed at competent disaster medical teacher development should be established together with a strengthened undergraduate program.

### 5.2. Teaching extent improves self-assessed disaster medical knowledge

The correlation between the amount of disaster medical education and self-assessed disaster medical knowledge must have an impact on Swedish educational policies. A national disaster medical curriculum can be developed based on the results of our study. Whether the current efforts by the Swedish National Board for Health and Welfare toward national curricula for dedicated undergraduate and postgraduate disaster medical education will improve the knowledge and preparedness of physicians and nurses in medical disaster response is unknown. Therefore, a follow-up of this study is recommended to assess the effectiveness of these centrally organized and government-driven efforts.

### 5.3. Military service improves self-assessed disaster medical knowledge

As we found military service to be an independent predictor of self-assessed disaster medical knowledge among both medical and nursing students, the current development toward a conscript army must be welcomed from a disaster preparedness perspective. We believe that the collaborative development of disaster medical content in military conscript training and undergraduate medical and nursing programs may create synergies between the defense and civilian educational systems. As these developments are an unprecedented opportunity for research future studies should investigate whether Sweden’s new conscript army will result in a stronger disaster medical workforce.

## Data availability statement

The raw data supporting the conclusions of this article will be made available by the authors, without undue reservation.

## Ethics statement

The studies involving human participants were reviewed and approved by Swedish Ethical Review Authority. The patients/participants provided their written informed consent to participate in this study.

## Author contributions

YR designed the study and collected the data. YR and JS performed the analysis and collaboratively wrote the paper. LR and FD critically revised the manuscript. All authors contributed to the article and approved the submitted version.

## Funding

This study was funded by the Swedish National Board of Health and Welfare (Socialstyrelsen, no. 2.7-3329/2023).

## Conflict of interest

The authors declare that the research was conducted in the absence of any commercial or financial relationships that could be construed as a potential conflict of interest.

## Publisher’s note

All claims expressed in this article are solely those of the authors and do not necessarily represent those of their affiliated organizations, or those of the publisher, the editors and the reviewers. Any product that may be evaluated in this article, or claim that may be made by its manufacturer, is not guaranteed or endorsed by the publisher.
