# Supplementary material for: Teaching extent and military service improve undergraduate self-assessed knowledge in disaster medicine: An online survey study among Swedish medical and nursing students
Source: Front Public Health. 2023 Mar 31;11:1161114. doi: 10.3389/fpubh.2023.1161114 (PMC10102457; doi:10.3389/fpubh.2023.1161114)
Supplement: Supplementary file 1 [file Image_1.pdf]

Hej!

Det fanns en tid, där svensk katastrofmedicinsk utbildning var en förebild för andra länder i världen. Tyvärr är den tiden över. Nedmonteringen av det civila försvaret har har drabbat även katastrofmedicin. Idag innehåller få läkar- och sjuksköterskeprogram obligatoriska katastrofmedicinska utbildningsmoment.

***Svenskt nationellt råd inom katastrofmedicin* skickar denna enkät till läkare och sjuksköterskor samt studenter på läkar och sjuksköterskeprogrammen för att skapa ett utgångsläge inför ett kommande utvecklingsarbete.**

Själva enkäten tar c:a 5 min. Inga personuppgifter kommer att inhämtas. Forskningshuvudman för projektet är Göteborgs universitet.

Vi tackar dig för din insats för katastrofmedicin i Sverige!

* 1. Samtycke

☐ Jag har tagit del av forskningspersonsinformationen och samtycker till att delta i enkäten om katastrofmedicinsk utbildning.

Demografi

2. Vilket kön har du?

- Kvinna
- Man
- Ickebinär
- Inget av ovanstående alternativ

3. När är du född?

4. I vilken region är du bosatt?

5. Är du studerande eller yrkesverksam?

- Läkarstuderande
- Sjuksköterskestuderande
- Läkare
- Sjuksköterska
- Annat

Läkare

Specifika frågor för examinerade läkare

6. Vid vilken universitet gick du läkarprogrammet?

7. När tog du läkarexamen?

8. Är du specialistläkare?

- Jag är specialistläkare
- Jag är ST-läkare
- Inget av ovanstående alternativ

9. Har du en chefsroll idag?

- Ja, med underställda chefer
- Ja, första linjens chef
- Nej

Specialistläkare eller ST-läkare

10. Inom vilken(a) specialitet(er)?

- [ ] Akutsjukvård
- [ ] Allergologi
- [ ] Allmänmedicin
- [ ] Anestesi och intensivvård
- [ ] Arbets- och miljömedicin
- [ ] Arbetsmedicin
- [ ] Barn- och ungdomsallergologi
- [ ] Barn- och ungdomshematologi och onkologi
- [ ] Barn- och ungdomskardiologi
- [ ] Barn- och ungdomskirurgi
- [ ] Barn- och ungdomsmedicin
- [ ] Barn- och ungdomsmedicinska specialiteter
- [ ] Barn- och ungdomsneurologi med habilitering
- [ ] Barn- och ungdomspsykiatri
- [ ] Beroendemedicin
- [ ] Bild- och funktionsmedicinska specialiteter
- [ ] Endokrinologi och diabetologi
- [ ] Geriatrik
- [ ] Gynekologisk onkologi
- [ ] Handkirurgi
- [ ] Hematologi
- [ ] Hud- och könssjukdomar
- [ ] Infektionssjukdomar
- [ ] Internmedicin
- [ ] Kardiologi
- [ ] Kirurgi
- [ ] Klinisk farmakologi
- [ ] Klinisk fysiologi
- [ ] Klinisk genetik
- [ ] Klinisk immunologi och transfusionsmedicin
- [ ] Klinisk kemi
- [ ] Klinisk mikrobiologi
- [ ] Klinisk patologi
- [ ] Klinisk neurofysiologi

- [ ] Kärlkirurgi
- [ ] Lungsjukdomar
- [ ] Medicinsk gastroenterologi och hepatologi
- [ ] Neonatologi
- [ ] Neurokirurgi
- [ ] Neurologi
- [ ] Neuroradiologi
- [ ] Njurmedicin
- [ ] Nuklearmedicin
- [ ] Obstetrik och gynekologi
- [ ] Onkologi
- [ ] Ortopedi
- [ ] Palliativ medicin
- [ ] Plastikkirurgi
- [ ] Psykiatri
- [ ] Radiologi
- [ ] Rehabiliteringsmedicin
- [ ] Reumatologi
- [ ] Rättsmedicin
- [ ] Rättspsykiatri
- [ ] Skolhälsovård (medicinska insatser i elevhälsan)
- [ ] Smärtlindring
- [ ] Socialmedicin
- [ ] Thoraxkirurgi
- [ ] Urologi
- [ ] Vårdhygien
- [ ] Äldrepsykiatri
- [ ] Ögonsjukdomar
- [ ] Öron-, näs- och halssjukdomar

Sjuksköterska

Specifika frågor till examinerade sjuksköterskor

11. Vid vilket lärosäte gick du sjuksköterskeprogrammet?

12. När tog du sjuksköterskeexamen?

13. Är du specialistsjuksköterska?

- Ja
- Nej

14. Har du en chefsroll idag?

- Ja, med underställda chefer
- Ja, första linjens chef
- Nej

Specialistsjuksköterska

15. När slutförde du din senaste specialistsjuksköterskeutbildning?

16. Inom vilken specialitet?

- [ ] Akutsjukvård (Akutsjuksköterska)
- [ ] Ambulanssjukvård (Ambulanssjuksköterska)
- [ ] Anestesisjukvård (Anestesisjuksköterska, kallas även narkossköterskor)
- [ ] Specialistsjuksköterska med inriktning mot hälso- och sjukvård för barn och ungdomar (Barnsjuksköterska)
- [ ] Diabetesvård (Diabetessjuksköterska)
- [ ] Distriktssköterska
- [ ] Företagshälsovård (Företagssjuksköterska)
- [ ] Hjärtsjukvård (Hjärtsjuksköterska)
- [ ] Infektionssjukvård (Infektionssjuksköterska)
- [ ] Intensivvård (Intensivvårdssjuksköterska)
- [ ] Kirurgisk vård (Kirurgisjuksköterska)
- [ ] Medicinsk vård (Medicinsjuksköterska)
- [ ] Onkologi (Onkologisjuksköterska)
- [ ] Operation (Operationssjuksköterska)
- [ ] Pallitativ vård (Palliativ sjuksköterska)
- [ ] Psykiatri (Psykiatrisjuksköterska)
- [ ] Skolsköterska
- [ ] Vård av äldre (Geriatrisjuksköterska)
- [ ] Inget av ovanstående alternativ

Katastrofmedicin på grundutbildning

17. Ingick katastrofmedicin i din grundutbildning?

- Ja, som obligatorisk kurs
- Ja, som valbar kurs
- Nej

18. Hur många timmar omfattade din katastrofmedicinska utbildning på läkar- eller sjuksköterskeprogrammet?

- mindre än 1 timme
- 1 till 2 timmar
- 3 till 5 timmar
- 1 heldag
- 2 till 4 heldagar
- 1 till 2 veckor
- mer än 2 veckor

19. Hur många timmar katastrofmedicinsk utbildning har du deltagit i efter din legitimering?

- mindre än 1 timme
- 1 till 2 timmar
- 3 till 5 timmar
- 1 heldag
- 2 till 4 heldagar
- 1 till 2 veckor
- mer än 2 veckor

20. Har du en master eller magisterexamen inom katastrofmedicin

- Ja
- Nej

21. Har du deltagit i katastrofmedicinska övningar?

- Ja, under grundutbildningen
- Ja, efter legitimation
- Nej

Läkarstuderande

22. Vid vilken universitet genomför du läkarprogrammet?

23. I vilken termin befinner du dig nu?

24. Har du erhållit utbildning i katastrofmedicin?

- ◯ Ja, obligatorisk kursmoment
- ◯ Ja, valbar kursmoment
- ◯ Nej

25. Hur många timmar katastrofmedicinsk utbildning har du fått?

26. Ingår katastrofmedicinska övningar i utbildningsmomenten?

- ◯ Ja
- ◯ Nej

Sjuksköterskestuderande

27. Vid vilket lärosäte går du sjuksköterskeprogrammet?

28. I vilken termin befinner du dig nu?

29. Har du erhållit utbildning i katastrofmedicin?

- Ja, obligatorisk kursmoment
- Ja, valbar kursmoment
- Nej

30. Hur många timmar katastrofmedicinsk utbildning har du fått?

31. Ingår katastrofmedicinska övningar i utbildningsmomenten?

- Ja
- Nej

Militär eller civil tjänstgöring

32. Har du tjänstgjort i Försvarsmakten?

- Ja
- Nej

33. När påbörjade du din militärtjänstgöring?

34. Har du haft en sjukvårdsbefattning under din tjänstgöring?

35. Har du en yrkesutbildning inom räddningstjänsten eller som polis?

- Nej
- Ja

36. Har du deltagit i utbildningar genom civila aktörer (Röda korset, MSB, EUCP, FN organ osv)?

- Ja
- Nej

Om ja, vilka kurser?

Självskattning

37. Bedöm följande uttalanden om din katastrofmedicinska kunskap.

	Instämmer helt	Instämmer i stor utsträckning	Neutral	Instämmer i liten utsträckning	Instämmer inte alls	ej tillämplig
Jag är väl införstådd i katastrofmedicinsk terminologi och juridik.	○	○	○	○	○	○
Jag är väl införstådd i katastrofmedicinsk organisation och ledning.	○	○	○	○	○	○
Jag har god kännedom om hur man hanterar ett stort skadeutfall som överlastar sjukvårdssystemet.	○	○	○	○	○	○
Jag är väl införstådd i hur katastroflarm och evakuering av sjukhus genomförs.	○	○	○	○	○	○
Jag kan bedöma nyttan med katastrofmedicinska insatser vid praktiska exempel.	○	○	○	○	○	○
Jag har kompetensen att använda triageprinciper i en prehospital kontext och på sjukhus.	○	○	○	○	○	○
Jag är väl införstådd i planering och genomförandet av en nödvändig evakuering.	○	○	○	○	○	○
Jag är väl införstådd i grundläggande behandlingsprinciper vid katastrofer (livräddande behandlingar, chockbehandling, ...).	○	○	○	○	○	○
Jag är väl införstådd i specifika behandlingsprinciper vid katastrofer (akuta kirurgiska operationer, behandling av brännskador, ...).	○	○	○	○	○	○

	Instämmer helt	Instämmer i stor utsträckning	Neutral	Instämmer i liten utsträckning	Instämmer inte alls	ej tillämplig
Jag är väl införstådd i katastrofmedicin efter olyckor med radioaktiv material och dekontaminering.	◯	◯	◯	◯	◯	◯
Jag är väl införstådd i hantering av farligt gods och olyckor, samt hantering av massförgiftningar med kemikalier och kemisk dekontaminering.	◯	◯	◯	◯	◯	◯
Jag är väl införstådd i de etiska principer för katastrofmedicin och prioriteringsprinciper i kris och krig.	◯	◯	◯	◯	◯	◯
Jag är väl införstådd i sjukdomar som förekommer efter katastrofer samt om behovet av psykologiskt krisstöd.	◯	◯	◯	◯	◯	◯
